# H/ACA snoRNAs and snoRNPs Dysregulation Links rRNA Modification to Glioblastoma Progression

**DOI:** 10.21203/rs.3.rs-8079210/v1

**Published:** 2025-12-11

**Authors:** Gabriela D. A. Guardia, Xiufen Lei, Christina Middle, Morgan Roos, Lea Damaschke, Sreenevash Ramesh, Stella Auth, Gabriel Lucas Fonseca, Nicole Acevedo, Frederico G. C. Oliveira, Nidhi Shah, Gary Schroth, Susanne Angelow, Andrew Brenner, Scott Kuersten, Pedro A. F. Galante, Luiz O. Penalva

**Affiliations:** Hospital Sírio-Libanês, São Paulo, SP; Children’s Cancer Research Institute, UT Health San Antonio, San Antonio, TX.; Illumina, Inc., San Diego, CA; Illumina, Inc., San Diego, CA; Children’s Cancer Research Institute, UT Health San Antonio, San Antonio, TX.; Children’s Cancer Research Institute, UT Health San Antonio, San Antonio, TX.; Children’s Cancer Research Institute, UT Health San Antonio, San Antonio, TX.; Children’s Cancer Research Institute, UT Health San Antonio, San Antonio, TX.; Children’s Cancer Research Institute, UT Health San Antonio, San Antonio, TX.; Hospital Sírio-Libanês, São Paulo, SP; Departamento de Bioquímica, Universidade de São Paulo, SP.; Illumina, Inc., San Diego, CA; Illumina, Inc., San Diego, CA; Illumina, Inc., San Diego, CA; Mays Cancer Center, UT Health San Antonio, San Antonio, TX.; Illumina, Inc., San Diego, CA; Hospital Sírio-Libanês, São Paulo, SP; Children’s Cancer Research Institute, UT Health San Antonio, San Antonio, TX.; Department of Cell Systems and Anatomy, UT Health San Antonio, San Antonio, TX.

**Keywords:** snoRNAs, scaRNAs, rRNA modification, snoRNPs, DKC1, Ampliseq

## Abstract

**Background.:**

Small nucleolar RNAs (snoRNAs) are critical players in ribosome biogenesis and have essential roles in rRNA processing and modification (2’O methylation and pseudo-uridylation). snoRNAs define which nucleotides get modified by guiding small nucleolar ribonucleoprotein complexes (snoRNPs) to specific positions in rRNA via base pairing. Altered snoRNA expression has been reported in diverse biological and pathological contexts. In cancer cells, the dysregulation of snoRNAs can alter the rRNA modification landscape, potentially affecting ribosome composition and translation. In aggressive tumors, such as glioblastoma (GBM), snoRNA profiling is crucial for expanding our understanding of ribosome biogenesis and identifying novel biomarkers and therapeutic targets.

**Methods.:**

We have developed an Ampliseq platform and analysis pipeline to measure the expression of 127 snoRNAs (mostly those containing H/ACA boxes) and Cajal body–specific RNAs (scaRNAs) and conducted a study in a panel of glioma stem cell (GSC), GBM, and normal neuronal lines/tissue.

**Results.:**

The results of our Ampliseq analysis identified snoRNAs and scaRNAs (snoRNAs/scaRNAs) that were differentially expressed in GBM and GSC cells compared to normal neuronal cells/tissue, as well as snoRNAs/scaRNAs associated with stemness and differentiation. SnoRNAs with elevated expression in GSC and GBM lines (snoRA38, snoRA51, snoRA71, and snoRA75) have been previously implicated in cancer development. Components of the H/ACA snoRNP complex, which regulate snoRNA processing and rRNA pseudouridylation, were also found to be overexpressed in GBM and showed decreased levels during neuronal differentiation. Notably, high expression of Dyskerin (DKC1)—the pseudouridylation enzyme and a key H/ACA snoRNP component—correlates with poor survival in patients with high-grade gliomas. Finally, we assessed the therapeutic potential of targeting snoRNAs in GBM. Knockdown of two upregulated snoRNAs, snoRA46 and snoRA75, using antisense oligonucleotides significantly impaired GBM cell growth.

**Conclusions:**

snoRNA/scaRNAs profiling revealed distinct alterations in snoRNA expression between glioblastoma and normal neuronal cells. These differences may contribute to the reprogramming of rRNA modification and ribosome composition in cancer cells. Moreover, our findings highlight the potential of antisense oligonucleotide-based targeting of overexpressed snoRNAs in GBM as a promising therapeutic strategy.

## INTRODUCTION

Ribosome biogenesis is a complex, multi-step process that generates and assembles the RNA and protein components of the 80S ribosome. Cancer cells are highly dependent on this process to sustain their increased and specialized protein demands (1–4). Consistent with this, altered expression of ribosomal proteins and regulators of rRNA transcription, processing, and modification has been linked to cancer development. Targeting ribosome biogenesis has therefore emerged as a promising therapeutic strategy, particularly in aggressive tumors such as glioblastoma (GBM) (3, 4). To improve the likelihood of clinical success, it is crucial to conduct focused studies to identify the RNA and protein factors driving rRNA alterations in each tumor type and to evaluate their roles in tumor progression and therapeutic response.

Ribosomal RNA (rRNA) modification is a key step in ribosome biogenesis; its impact on RNA-RNA and RNA-protein interactions can ultimately alter ribosome composition and influence translation. Growing evidence suggests that ribosomes are heterogeneous, and “specialized” ribosomes have been described in different scenarios (5–10). A recent study, employing ribosome ratio-omics (RibosomeR) to investigate 80S ribosome proteomics data, identified major variations in ribosome protein stoichiometry across various biological samples, including differences between tissues, developmental stages, and pathological states (9). In cancer cells, changes in ribosome composition can enhance and/or tailor translation to promote the production of proteins involved in tumor progression, therapy resistance, and metastasis (8).

RNA-protein complexes called snoRNPs regulate the two main types of rRNA modifications, 2’O methylation and pseudo-uridylation. 2’O methylation occurs in all four nucleotides and is catalyzed by Fibrillarin (FBL) while pseudo-uridylation (the conversion of uridine into pseudo-uridine, Ψ) is catalyzed by dyskerin (DKC1) (10). DKC1 driven pseudo-uridylation has two additional regulatory impacts. Small Cajal body-specific RNAs (scaRNAs) guide DKC1 to pseudo-uridylate specific nucleotides in small nuclear RNAs (snRNAs). snRNAs are essential components of the spliceosome and have important functions in splice site recognition and selection (11). DKC1 also binds the telomerase RNA (TERC) and modulates its activity (12).

Small nucleolar RNAs (snoRNAs) define which nucleotides in rRNA get modified by guiding snoRNPs to specific positions in rRNA via base pairing. More than 500 snoRNAs, ranging from 60 to 300 nucleotides, have been cataloged in the human genome. There are two classes of snoRNAs: C/D box snoRNAs are implicated in 2’-O-methylation, while snoRNAs with H/ACA boxes regulate pseudo-uridylation (Ψ) (13, 14). Most expressed snoRNAs are embedded in introns of lncRNAs and protein-coding genes, including ribosomal proteins and rRNA transcription/processing regulators. However, snoRNA expression levels do not always correlate with those of their host genes, reflecting the influence of specific sequence features and differences in snoRNA maturation (15). Alterations in snoRNA expression have been observed in various disease states and cancers, with several snoRNAs contributing to cancer-relevant phenotypes and tumor growth (16).

Despite their biological relevance and potential as biomarkers or therapeutic targets, studies on snoRNA and scaRNA profiling remain very scarce. The lack of specific sequencing approaches for snoRNA/scaRNA analysis is certainly a barrier. We developed an Ampliseq panel and analysis pipeline to evaluate H/ACA box snoRNAs and scaRNAs specifically, and conducted the first comprehensive study of snoRNAs and scaRNAs in GBM versus normal cells. Results led to the identification of multiple snoRNAs/scaRNAs aberrantly expressed in tumor cells, defined a snoRNA/scaRNA signature for glioma stem cells, and identified snoRNAs/scaRNAs associated with stemness and differentiation. Our study also demonstrated the potential use of antisense oligonucleotides (ASOs) to target aberrantly expressed snoRNAs in GBM as a potential therapeutic strategy.

## METHODS

### Cell culture

Human glioblastoma cell line U251 was obtained from Uppsala University (Uppsala, Sweden), while the T98G and LN229 cell lines were obtained from the American Type Culture Collection. Human astrocyte cells (Cat# 1800) were obtained from ScienCell Research Laboratories (Carlsbad, CA, USA). Glioblastoma Stem Cell (GSC) lines 3565 and 1919 were gifts from Drs. Jeremy Rich, Christopher Hubert, and Ichiro Nakano (17, 18). GSC lines 031417, 040909, and 040815 were established by Dr. Andrew Brenner’s lab. Sareddy. Neural Progenitor Cells (NPCs) were purchased from Axol Bio (Cat# ax0011). The U251, T98G, LN229, and HEK293T cell lines were cultured in DMEM medium (HyClone; Cat# SH30243.01) supplemented with 10% Fetal Bovine Serum (FBS) (Corning; Cat# 35015CV) and 1% penicillin/streptomycin (Gibco; Cat# 10378016). Human astrocyte cells were cultured in DMEM-F12 media (Thermo Fisher Scientific, Cat# 11320033). supplemented with 10% FBS (Corning) and 1% penicillin/streptomycin (Gibco). All GSCs and NPCs were cultured in Neurobasal-A medium supplemented with B27, glutamine, sodium pyruvate, 20 ng/mL of both epidermal growth factor (EGF) (Thermo Fisher Scientific) and basic fibroblast growth factor (bFGF) (PeproTech). All cells were maintained in a humidified incubator at 37°C with 5% CO_2_. For routine passaging, cells were harvested by using 0.05% Trypsin/0.53 mM EDTA in HBSS (Corning, Cat# 25–051-Cl) and replated for continued culture. For subsequent experiments, cells were harvested and counted with the Countess automated cell counter (Invitrogen) using trypan blue, then plated at specific densities for transfection and the various assays described below.

### Cell transfection

For transient gene knockdown, cells were transfected with small interfering RNA (siRNA) or antisense oligonucleotides (ASOs) using Lipofectamine RNAiMAX (Invitrogen, Carlsbad, CA; Cat# 13778150) and OptiMEM (Thermo Fisher Scientific, Cat# 31985070). Transfected cells were harvested after 72 hours for RNA analysis or incubated for other assays as described below. siRNAs were obtained from Dharmacon (Lafayette, CO) and Millipore-Sigma (Burlington, MA). ON-TARGETplus non-targeting siRNA (Dharmacon; Cat# D-001810-01-05) was used as a negative control. The DKC1 siRNA (siDKC1, SASI_Hs01_00195836) was obtained from Millipore-Sigma. ASOs were purchased from Millipore Sigma.

### Cell proliferation assay

U251-GFP cells were harvested, counted, and seeded at a density of 1200 cells/well into 96-well plates. After transfection with ASOs, plates were transferred to a live-cell imaging system (IncuCyte, Essen BioScience). Cell proliferation was subsequently monitored for 72 consecutive hours, with images acquired and cells counted every four to six hours.

### MTS assay

U251 cells were harvested, counted, and seeded at a density of 1200 cells/well into 96-well plates. Plates were incubated at 37°C for 72 hours after transfection with ASOs as described above. Next, 20μl of MTS mixture (1,000μl MTS and 50μl PMS) of MTS solution (Promega) was added to each well, and samples were incubated at 37°C for 60 minutes. Optical density was measured at absorbance 490nm with a Synergy HT microplate reader (BioTek).

### Co-culture assay

U251 GFP cells (800 cells/well) and human astrocyte cells (1000 cells/well) were harvested, counted and seeded into a 96-well plate. ASO transfection, as described above, was performed simultaneously with cell seeding, using a final ASO concentration of 40 nM. Cell proliferation and morphology were subsequently monitored for five consecutive days using the IncuCyte automated microscope system (Essen Bioscience), with images acquired every six hours. 2×2 montage images per well were manually analyzed using ImageJ. A digital grid was overlaid, and the number of astrocytes within four fields was counted.

### RNA preparation

Total RNA was extracted from cells using TRIzol^™^ reagent (Thermo Fisher Scientific, Grand Island, NY; Cat# 15596018), following the manufacturer’s instructions. RNA concentration and purity were determined spectrophotometrically using a NanoDrop 8000 (Thermo Scientific). Only RNA samples with an acceptable A260/A280 ratio were used for subsequent steps. The RNA was stored at −80°C until further use. For complementary DNA (cDNA) synthesis, 300 ng of total RNA from each sample was reverse transcribed using the High-Capacity cDNA Reverse Transcription Kit (Thermo Fisher Scientific; Cat# 4368814) according to the manufacturer’s protocol. The resulting cDNA was stored at −20°C until further use.

### Quantitative Real-Time PCR

To validate the knockdown efficiency of the DKC1 siRNA and the ASOs against snoRA46 and snoRA75, qRT-PCR was performed on transfected U251 cells. Gene expression was normalized to GAPDH, a housekeeping gene, using the ddCT method. For all qRT-PCR experiments, the PowerUp SYBR Green Master Mix (Applied Biosystems) was used. Sequences of qRT-PCR primers are listed in **Table S1**, and sequences of ASOs are presented in **Figure S1**.

### RNA sample preparation and RNA-seq

Total RNA from transfected U251 cells (siRNA control vs. siDKC1) was extracted using the TRIzol reagent (Invitrogen, Carlsbad, CA) according to the manufacturer’s instructions. Libraries for RNA sequencing were prepared using the TruSeq RNA Library Preparation Kit (Illumina, San Diego, CA), following the manufacturer’s instructions. The prepared libraries were then sequenced at UT Health San Antonio Genomic Facility. All experiments were performed in triplicate.

### Amplicon sequencing (Ampliseq)

The snoRNA/scaRNA Ampliseq panel was designed using the HUGO Gene Nomenclature Committee (HGNC; database (19), which contains 564 entries. Due to design constraints on the length of gene entries (genes < 125 nucleotides were omitted), 213 multiplex primer pairs were designed for 127 snoRNA/scaRNA genes (**Table S2**). The RNA sequencing (RNAseq) libraries were constructed using the AmpliSeq for Illumina On-Demand, Custom, and Community Panels reference guide, following the standard workflow listed in the protocol. Briefly, Total RNA (~ 10–100 ng) was reverse transcribed with random primers, then combined with the AmpliSeq snoRNA/scaRNA multiplex primer pairs and amplified by PCR for 17 cycles. The amplicons were partially digested with the FuPa enzyme and ligated to the AmpliSeq CD Indexes for Illumina. The samples were purified with Illumina Purification Beads (IPB) and PCR-amplified a second time for 7 cycles using generic (P5/P7) primers standard to all amplicons. The samples were then purified for a second time prior to quantification using the Qubit high-sensitivity assay. The samples were then normalized and pooled for sequencing. The libraries were then sequenced on a NextSeq 550 or NextSeq 2000 instrument at 2 × 150 paired-end reads (**Figure S2A**).

### Ampliseq analysis

AmpliSeq RNA sequencing reads were aligned to the human reference genome (GRCh38/hg38) using BWA-MEM with default parameters (https://github.com/lh3/bwa, Accessed 23 October 2025). Only properly paired reads were retained using SAMtools with parameter -f 0×0002 (20). To remove ambiguously mapped reads, read pairs exhibiting identical primary (AS) and suboptimal (XS) alignment scores for both mates were filtered out using local scripts. This filtering step ensured that only uniquely aligned read pairs were retained for downstream quantification. Gene-level read counts were quantified using HTSeq-count (parameters: -m intersection-strict and -s no) (21), based on gene annotations from the NCBI RefSeq (hg38). Differential expression analyses of snoRNA genes between sample groups were performed using DESeq2 (22). Genes with |log_2_FC| ≥ 0.6 and a false discovery rate (FDR) < 0.05 were considered differentially expressed.

### Splicing analysis

To evaluate the impact of DKC1 knockdown on RNA splicing, RNA-Seq reads were pseudo-aligned to the GENCODE human reference transcriptome (version 36) (Frankish et al., 2021) using Kallisto (version 0.48) with default parameters and 100 bootstrap replicates (23). Normalized expression values (TPM) were then used as input to SUPPA2 (v. 2.3), with default parameters (24), to detect differential alternative splicing events. Alternative splicing events in each gene were generated with the “generateEvents” function and classified as skipping exon (SE), alternative 5’ (A5S) and 3’ (A3S) splice sites, mutually exclusive exons (MX), or retained intron (RI). Events were considered significant when |ΔPSI| >0.1 and False Discovery Rate (FDR) adjusted p-values < 0.05.

### RNA-Seq analyses of public datasets

To investigate the expression profiles of genes involved in H/ACA snoRNA processing and rRNA modification, as well as snoRNA host genes in healthy brain and glioma samples, we obtained publicly available gene expression datasets (TPM-normalized) from the GTEx (V8) (https://www.gtexportal.org/, Accessed 23 October 2025) and TCGA projects (https://portal.gdc.cancer.gov/, Accessed 23 October 2025), respectively. Normal (frontal cortex) tissue expression data (n = 464) were retrieved from GTEx, while data from lower-grade gliomas (LGG grade II, n = 249; LGG grade III, n = 265) and glioblastoma (GBM, IDH1/2 wild-type, n = 152) were obtained directly from TCGA. Boxplots were generated using the ggplot2 package (v. 4.0.0) in R, and statistical differences between groups were assessed using the Mann–Whitney U test.

For high-grade gliomas, we evaluated the impact of DKC1 gene expression on overall survival by stratifying patients into high- and low-expression groups based on the median TPM value. Survival analyses were performed using the survival (v. 3.8.3), survminer (v. 0.5.1), and forestmodel (v. 0.6.2) R packages. Multivariate analyses were conducted to adjust for tumor histology (GBM or LGG grade III).

To compare DKC1 expression levels across multiple tumor types, we analyzed publicly available gene expression data (TPM-normalized) from the GEPIA2 web platform (http://gepia2.cancer-pku.cn, Accessed 23 October 2025), which integrates GTEx and TCGA datasets. Only tumor types displaying significantly different expression levels between tumor and normal tissues (p < 0.05) were included: CESC (306 tumor and 13 normal samples), CHOL (36 tumor and 9 normal), COAD (275 tumor and 349 normal), DLBC (47 tumor and 337 normal), LUSC (486 tumor and 338 normal), READ (92 tumor and 318 normal), STAD (408 tumor and 211 normal), and THYM (118 tumor and 339 normal).

To assess DKC1 expression changes during cortical development, we obtained TPM-normalized gene expression data from the Cortecon database (25). Boxplots were generated using ggplot2 (v. 4.0.0), and statistical significance was assessed by Mann–Whitney U tests.

Finally, to evaluate differences in DKC1 expression levels in neuroblastoma BE(2)C cells between day 0 and day 7 of ATRA-induced differentiation, we used RNA-Seq data from a previous study (26). First, RNA-Seq reads were pseudoaligned to the human reference transcriptome (GENCODE v36, https://www.gencodegenes.org/, Accessed 23 October 2025) using Kallisto (v. 0.48) (23). Gene-level count data were then obtained using the tximport R package (v. 1.26.1) and differential gene expression analysis was performed with DESeq2 (v. 1.38.3) (22).

## RESULTS

### Expression of snoRNAs/scaRNAs is altered in glioblastoma.

**Figure 1:**
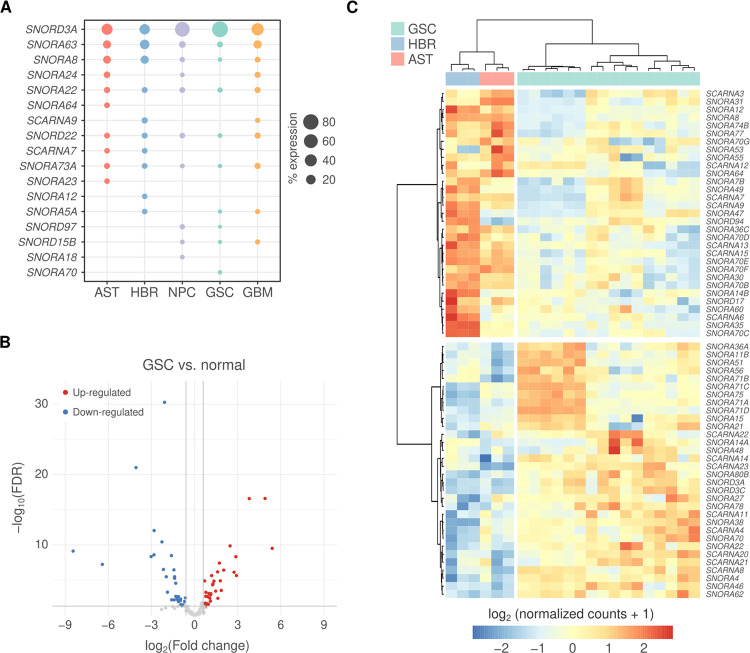
snoRNAs differentially expressed in glioma stem cells vs. normal brain. **A)** The topmost expressed snoRNAs in astrocytes (AST), normal brain (HBR), neural progenitor cells (NPC), glioma stem cells (GSC), and glioblastoma (GBM) cells. **B)** Volcano plot showing differentially expressed snoRNAs in GSCs vs. normal cells (AST/astrocytes and HBR/human brain). **C)** Heatmap showing differentially expressed snoRNAs in GSCs vs. normal cells (AST and HBR).

We devised an Ampliseq panel and analysis pipeline (**Figure S2**) to specifically evaluate the expression of 127 snoRNAs (primarily those containing H/ACA boxes) and scaRNAs. Among the 77 rRNA pseudouridylation sites with known guiding snoRNAs, our Ampliseq platform covers snoRNAs responsible for guiding 67 of these modifications (27, 28). We profiled their expression in glioblastoma cells, glioma stem cells (GSC), neuronal precursor cells (NPCs), astrocytes, and the normal human brain. In all samples, we observed that more than 50% of all detected snoRNA and scaRNA expression corresponds to a group of 10–12 snoRNAs. Notably, snoRD3A, snoRA63, snoRA8, snoRA22, snoRD22, and snoRA73A showed consistently high expression across all samples (Fig. 1A). From this group, snoRD3A, snoRA63, snoRA73A, and snoRD22 participate in specific steps of rRNA processing, while snoRA8 and snoRA22 are implicated in rRNA modification. Their markedly higher expression compared to other snoRNAs suggests that they may have additional regulatory roles. For instance, snoRD3A and snoRA73 interact with chromatin and bind numerous RNA species (29–32). Apart from snoRD3A, all highly expressed snoRNAs are located in the introns of host genes, present on distinct chromosomes (**Table S3**).

**Figure 2:**
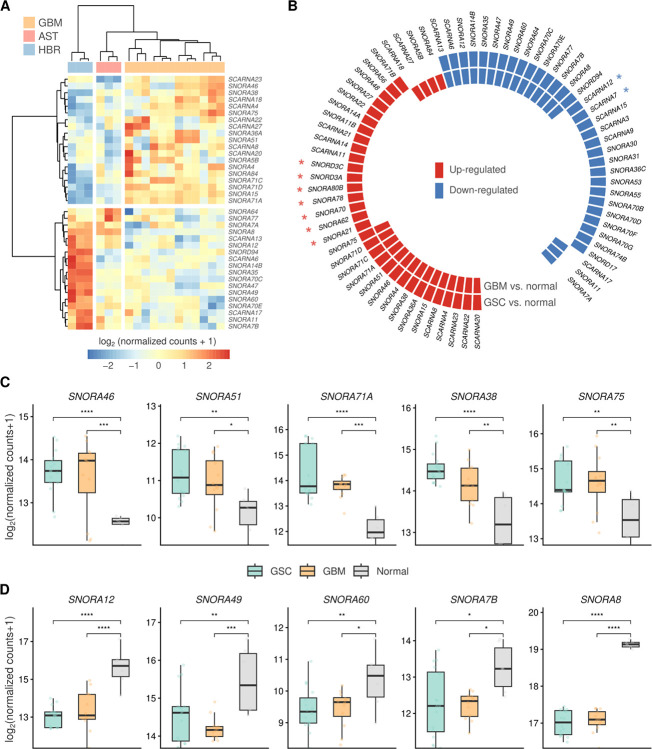
snoRNAs/scaRNAs differentially expressed in glioblastoma cells vs. normal brain. **A)** Heatmap showing differentially expressed snoRNAs in GBM cells vs. normal (AST and HBR). **B)** Circos plot showing differentially expressed snoRNAs/scaRNAs in GBM and/or GSC vs. normal brain (astrocyte and HBR). * snoRNAs showing differential expression in GBM cells, but not statistically significant. **C-D)**Boxplots showing expression levels of snoRNAs **(C)** up-regulated or **(D)** down-regulated both in GBM and GSC vs. normal brain. Statistical differences were assessed by DESeq2 p-values (* < 0.05, ** < 0.01, *** < 0.001, **** < 0.0001).

To identify snoRNAs/scaRNAs with altered expression in glioblastoma, we conducted two comparative analyses. First, we compared GSCs against neuronal cells and tissue (astrocytes and normal brain), which revealed two distinct groups of up- and down-regulated snoRNAs and scaRNAs in GSCs (Fig. 1B–C). Second, we compared GBM cell lines against astrocytes and normal brain (Fig. 2A). By integrating these two analyses, we identified snoRNAs/scaRNAs that were consistently altered in both GSCs and GBM lines, as well as those with cell-type-specific alterations, either exclusive to GSCs or to GBM cells (Fig. 2B). Representative box plots of snoRNAs displaying the most prominent expression differences in both GSCs and GBM lines are shown in Fig. 2C–D.

We further analyzed NPCs in comparison with astrocytes/normal human brain tissue. Thirteen snoRNAs/scaRNAs were determined to be upregulated in both NPCs and GSCs in comparison to astrocytes and the normal human brain, defining a stemness-associated subset. On the other hand, eleven snoRNAs/scaRNAs showed increased expression in astrocytes/normal brain relative to NPCs and GSCs, establishing a subset linked to differentiation. Nineteen upregulated and twenty downregulated snoRNAs/scaRNAs were observed exclusively in the GSC analysis (**Figure S3**).

**Figure 3:**
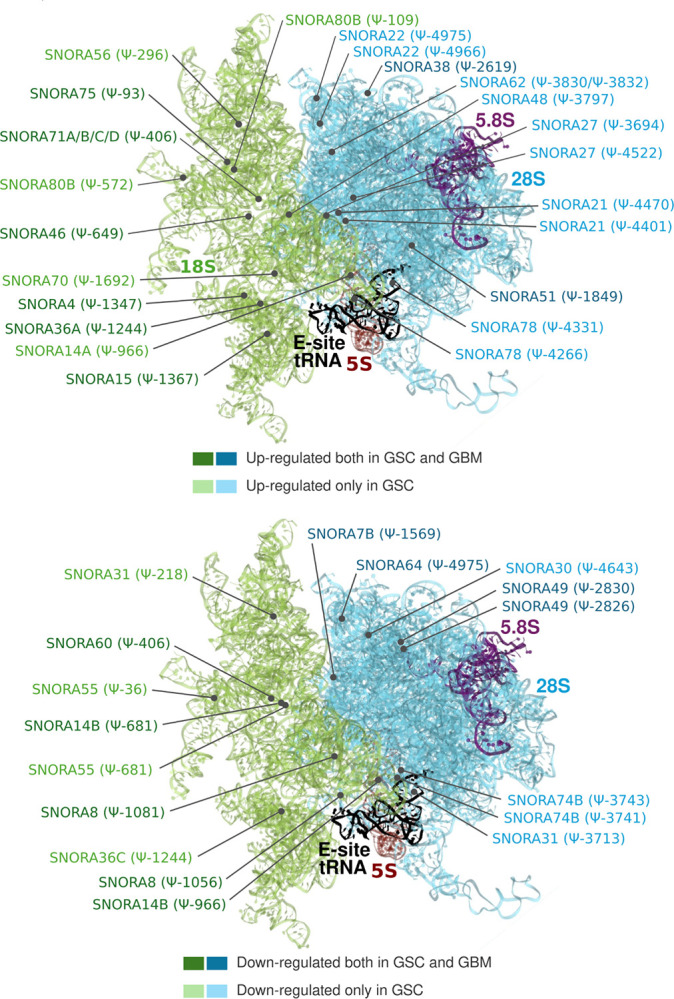
Differentially expressed snoRNAs in GSC and GBM lines and location of pertinent pseudo-uridylation sites in the 80S human ribosome according to Piekna-Przybylska et al., 2008 (33).

Differential snoRNA expression can alter the rRNA modification profile, consequently affecting ribosome assembly and composition. In Fig. 3 and **Table S4**, we show the snoRNAs displaying altered expression in GSC and/or GBM lines and their respective pseudo-uridylation sites in 18S and 28S rRNAs in the context of ribosome structure.

### snoRA46 and snoRA75 knockdown affected the growth of glioblastoma cells

**Figure 4:**
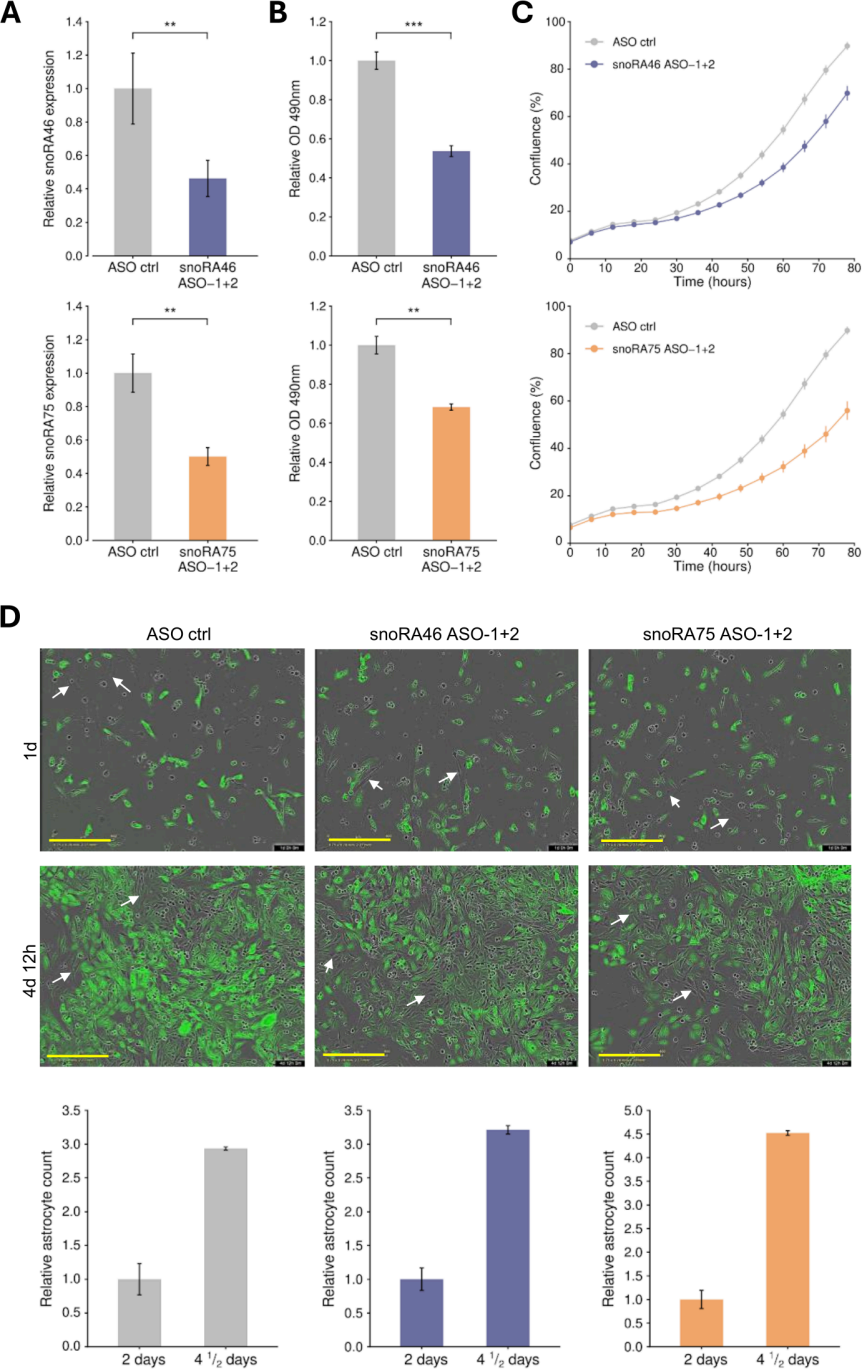
Knockdown of snoRA46 and snoRA75 affects glioblastoma cells. U251 **A)** Differences in snoRA46 and snoRA75 expression levels measured by qRT-PCR in control and ASO-transfected cells. **B)** Knockdown of snoRA46 and snoRA75 in U251 cells decreased viability (MTS assays). Data were analyzed with Student’s t-test and presented as the mean ± standard deviation. *** = p ≤ 0.001. **C)** U251 proliferation across time was followed with the Incucyte automated system. A decrease in snoRA46 and snoRA75 levels impaired cell proliferation. **D)** U251 cells (GFP labeled) and astrocytes were co-cultured and treated with control, snoRA46, or snoRA75 ASOs. Images show that treatment with snoRA46 or snoRA75 ASOs affected the proliferation of U251 (green cells), corroborating the Incucyte results. White arrows highlight astrocytes. Yellow bars = 400mM. Bar-graphs (bottom) show relative astrocyte counts in days 2 and 4 ½ and indicate that they were not affected by treatment with snoRA46 or snoRA75 ASOs.

Targeting aberrantly expressed snoRNAs with ASOs is an effective alternative for impairing the growth of cancer cells (34–36). We designed ASOs to knock down the expression of two snoRNAs, snoRA46 and snoRA75, which are upregulated in GSC and GBM cells, as well as in GBM tumors. Two ASOs were combined to increase knockdown levels. snoRA46 and snoRA75 knockdown affected the proliferation of U251 cells according to Incucyte analysis and cell viability (MTS assay). Additionally, in a co-culture assay, we demonstrated that transfection with snoRNA ASOs inhibited the growth of U251 cells (GFP-labeled) but had no effect on astrocytes (Fig. 4).

#### Components of the snoRNP H/ACA are highly expressed in glioblastoma.

**Figure 5:**
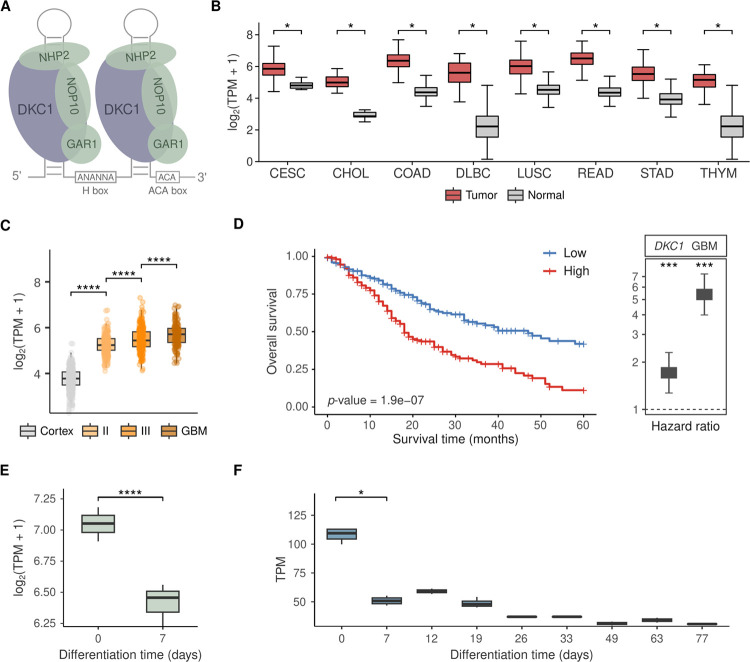
DKC1 is highly expressed in glioblastoma and associated with poor survival in high-grade glioma. **A)** Representation of H/ACA snoRNP. **B)**Expression levels of DKC1 in distinct tumor types vs. corresponding normal tissue obtained from TCGA and GTEx, respectively (data source: GEPIA2 database (37). Abbreviations: Cervical squamous cell carcinoma and endocervical adenocarcinoma (CESC), Cholangiocarcinoma (CHOL), Colon adenocarcinoma (COAD), Lymphoid Neoplasm Diffuse Large B-cell Lymphoma (DLBC), Lung squamous cell carcinoma (LUSC), Rectum adenocarcinoma (READ), Stomach adenocarcinoma (STAD), Thymoma (THYM). **C)** Expression levels of DKC1 in normal cortex, gliomas grade II and III, and glioblastoma. Statistical differences were assessed by Mann–Whitney U tests (p-value * < 0.05, ** < 0.01, *** < 0.001, **** < 0.0001). **D)** Survival curves of high-grade glioma patients with high/low expression of DKC1 and multivariate analysis adjusted by GBM histology. Statistical differences were assessed by log-rank tests. **E)** DKC1 expression levels in BE2C neuroblastoma cells on day 0 and day 7 of ATRA-induced differentiation. Statistical differences were evaluated by DESeq2 p-values (**** < 0.0001).**F)** Expression levels of DKC1 during cortex development according to Cortecon (25). Statistical differences were assessed by Mann–Whitney U tests (p-value * < 0.05, ** < 0.01, *** < 0.001, **** < 0.0001). Non-significant comparisons are not shown.

The snoRNPs in charge of rRNA pseudo-uridylation are composed of four main proteins: DKC1, NOP10, NHP2, and GAR1, with DKC1 being the enzyme catalyzing the conversion of uridine into pseudo-uridine – Fig. 5A. DKC1 expression levels are elevated in numerous tumor types in relation to their normal counterparts – Fig. 5B. Expression analysis using data from GTEx for normal brain and TCGA for glioma indicated that DKC1 and other members of the snoRNP H/ACA show increased expression in GBM in comparison to normal brain (cortex) and low-grade glioma (LGG or grade II) – Fig. 5C, **Figure S4**. Interestingly, snoRA36A, which is located in DKC1 intron 8, is also upregulated in GBM. Survival analysis using TCGA’s high-grade glioma (Grades 3 and 4) patient data showed that high DKC1 expression correlates with poor survival – Fig. 5D. DKC1 also shows expression alterations during neuronal differentiation. Neuroblastoma BE(2)C cells treated with retinoic acid (ATRA) to induce differentiation (26) showed a decrease in DKC1 levels after 7 days– Fig. 5E. Analysis of the Cortecon dataset (25) indicated a similar trend, with DKC1 showing high expression in NPCs and a drastic decrease during cortical development – Fig. 5F. These results corroborate the association between increased DKC1 expression and the GBM poorly differentiated state.

### Factors influencing snoRNA expression changes in glioblastoma and glioma stem cells

**Figure 6:**
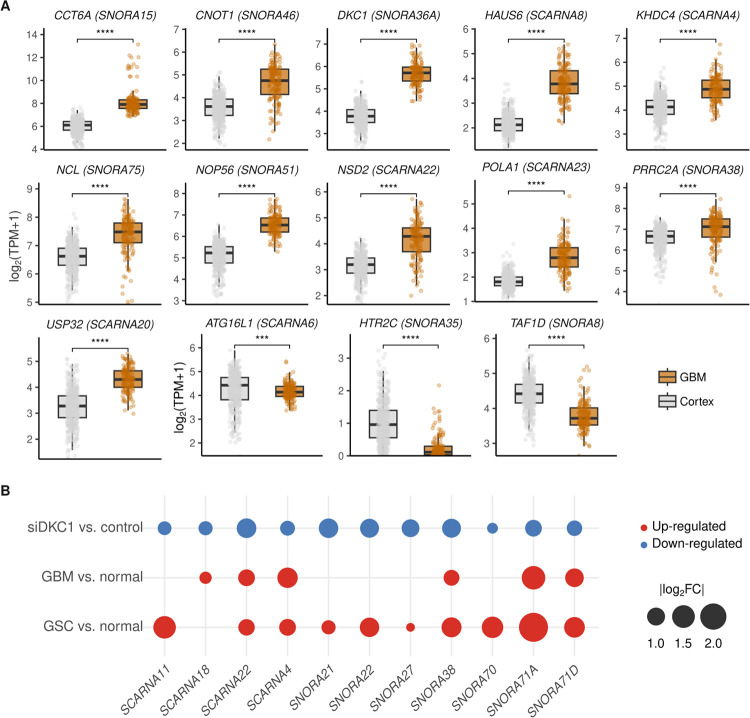
Potential factors driving snoRNAs and scaRNAs differential expression. **A)** Expression of host genes of differentially expressed snoRNAs/scaRNAs in GBM vs. normal brain. Only host genes displaying expression differences that resemble what has been observed for their respective associated snoRNA/scaRNA are represented. Statistical differences were assessed by Mann–Whitney U tests (p-value * < 0.05, ** < 0.01, *** < 0.001, **** < 0.0001). **B)**Bubbleplots with results of an RNAseq analysis showing snoRNAs/scaRNAs upregulated in GSC/GBM lines whose expression decreased after partial DKC1 knockdown in U251 cells. |log_2_FC| values are shown only for snoRNAs with FDR < 0.05.

Several factors contribute to the observed differences in snoRNA expression levels, including their processing from host transcripts, interactions with associated proteins, and intrinsic sequence and structural features (38, 39). Although most snoRNAs/scaRNAs are processed from introns of host genes, the expression levels of the host dictate the levels of its associated snoRNA/scaRNA. Among snoRNAs with decreased expression in GSC and GBM lines, snoRA12, snoRA14B, snoRA35, snoRA47, snoRA49, and snoRA55 are preferentially expressed in neuronal cells. Interestingly, snoRA35 and its host gene, HTRC2, are exclusively expressed in neuronal cells (13). snoRA35 is predicted to pseudo-uridylate the nucleotide 566 in 18S and could drive an essential difference between neuronal and tumor cells. All 29 snoRNAs/scaRNAs identified as differentially expressed in GSC and GBM cells are located within introns. We performed an expression analysis of GBM vs. normal brain to determine in which cases snoRNA expression levels are potentially influenced by their host genes. Results indicate that 14 host genes display significant differences in expression between brain and GBM, resembling what was observed for their associated snoRNAs/scaRNAs – Fig. 6A.

DKC1 is implicated in the processing of all H/ACA box-containing snoRNAs and scaRNAs and pseudo-uridylation of all sites in rRNA. However, certain snoRNAs and modification sites appear more “sensitive” to DKC1 mutations or changes in its expression levels, as observed in samples from dyskeratosis patients and cancer cells (40–44). We inquired whether high DKC1 expression observed in GBM/GSC cells could increase the levels of specific snoRNAs/scaRNAs. Ampliseq analysis determined that a partial reduction in DKC1 levels in U251 cells decreased the levels of several snoRNAs/scaRNAs determined to be upregulated in GSC and/or GBM cells, including snorA21, snoRA22, snoRA27, snoRA38, snoRA70, snoRA71A, snoRA71D, scaRNA4, scaRNA11, scaRNA18, and scaRNA22 – Fig. 6B and **Table S5**.

#### scaRNAs, pseudouridylation of snRNA, and splicing.

**Figure 7:**
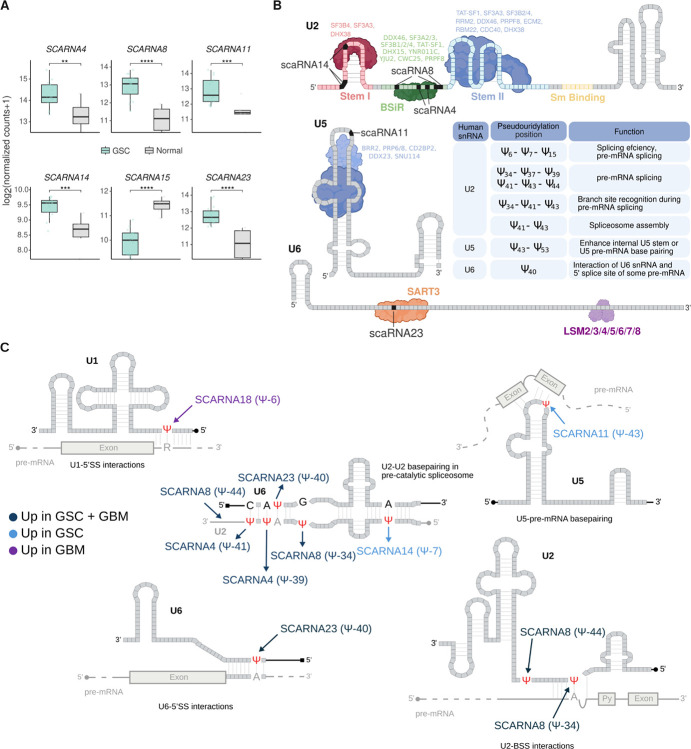
scaRNAs differentially expressed in GSCs and snRNA pseudo-uridylation. **A)** Boxplots showing expression levels of up-regulated or down-regulated scaRNAs in GSC vs. normal (brain and astrocytes). Statistical differences were assessed by DESeq2 p-values (* < 0.05, ** < 0.01, *** < 0.001, **** < 0.0001). **B)** Representation of relevant RNA-RNA interactions, showing pseudo-uridylation sites and the implicated scaRNAs. Table showing the regulatory relevance of pseudo-uridylation sites. **C)**Representation of U2, U5, and U6 snRNPs showing RNA-protein interactions, pseudo-uridylation sites, and the implicated scaRNAs.

Pseudo-uridylation also occurs in snRNAs and is driven by snRNPs containing DKC1 and scaRNAs. snRNAs are critical components of the spliceosome and are responsible for the recognition and selection of splice sites via base pairing. Pseudo-uridylation of snRNAs occurs at critical nucleotide positions, relevant for RNA-RNA and RNA-protein interactions (45, 46). We determined that several scaRNAs implicated in snRNA pseudo-uridylation at sites that affect RNA-RNA interactions show increased expression in GSC and GBM cells. The most relevant ones include scaRNA4, scaRNA8, scaRNA11, scaRNA14 and scaRNA23 - Fig. 7A–B. Alterations in scaRNAs have been observed in congenital heart disease (Tetralogy of Fallot) and shown to affect the splicing of RNAs critical for heart development (47). In cancer cells, scaRNA15 influences the splicing of transcripts encoding chromatin and transcriptional regulators, thereby impacting the expression and function of *ATRX* and *TP53* (48). Therefore, the alterations in DKC1 and scaRNA expression observed in tumor cells could ultimately contribute to their splicing profile and constitute a new route implicated in GBM development. In agreement, we determined that a partial DKC1 knockdown affected the splicing profile of U251 GBM cells (**Figure S5 and Table S6**).

## DISCUSSION

Using a dedicated Ampliseq platform, we performed the first snoRNA profiling in GBM and GCS cells, identifying sets of snoRNAs that were up- and down-regulated in comparison to normal neuronal cells/tissue and associated with stemness and differentiation. Several snoRNAs that are elevated in GSC and GBM lines have previously been implicated in cancer development. We highlight snoRA38, snoRA51, snoRA71, and snoRA75. snoRA38 is upregulated in breast cancer, and its expression levels correlated with tumor size, lymph node metastasis, and TNM stage (49). snorA38 and snoRA71 were identified as part of a 20 snoRNA/scaRNA signature associated with breast cancer brain metastases (50). Increased expression of snoRA38 and snoRA75 was observed in colon cancer metastasis to the liver (51). High snoRA51 has been observed in different tumor types. In breast cancer, increased snoRA51 expression was connected to worse prognosis, overall survival, and disease-free survival. In addition, snoRA51 enhanced cancer stem cell-like properties via the RPL3/NPM1/c-MYC pathway (52). snoRA51 is also upregulated in colon cancer and hepatocellular carcinoma (HCC) and is defined as a potential biomarker (53, 54). SnoRA75 is among a group of 12 snoRNAs that show significant correlation with tumor microenvironment immune infiltration in melanoma (55). It is also part of a 14 snoRNA signature that can significantly stratify AML patients into high- and low-risk groups (56). Five copies of snoRA71 (snoRA71, snoRA71A, snorA71B, snoRA71C, and snoRA71D) are located in various introns of SNHG17 gene. They share strong sequence similarity and are all implicated in pseudo-uridylation of nucleotide 406 in 18S rRNA. snoRA71 is highly expressed and has been identified as a prognostic marker of lung cancer (57, 58), hepatocellular carcinoma (HCC) (59), multiple myeloma (60), and colorectal cancer (61). SnoRA71 knockdown affected proliferation, migration, invasion, and tumor growth (58, 61, 62). A Pan-cancer study using TCGA small RNA-seq data from 31 cancer types (excluding glioblastoma) identified 46 clinically relevant snoRNAs (63), including snoRA71, which showed alterations in at least 12 different tumor types. Interestingly, it has recently been demonstrated that snoRA71 binds multiple chromatin sites, suggesting additional regulatory roles (31).

Several factors can contribute to differences in snoRNA and scaRNA levels (15, 38). The majority of snoRNAs and scaRNAs analyzed in our study are located in introns of host genes. We investigated the expression of 29 host genes associated with snoRNAs/scaRNAs in GBM/GSC lines. Fourteen host genes show expression differences between normal brain and GBM that resemble those observed for their respective associated snoRNA/scaRNA. Therefore, in these cases, the host genes are likely relevant contributors to the expression levels of their pertinent snoRNA/scaRNA.

Dyskerin (DKC1) is part of a protein complex involved in the processing of H/ACA snoRNAs and scaRNAs. It also catalyzes pseudo-uridylation in rRNA and snRNA, guided by H/ACA snoRNAs or scaRNAs, respectively (64, 65). Mutations in DKC1 cause dyskeratosis congenita, a multi-organ syndrome. In these patients, reduced pseudo-uridylation at specific nucleotides in 28S has been observed. These changes could compromise rRNA-ribosomal protein interactions, destabilizing the ribosome (40). Conversely, DKC1 is often highly expressed in cancers, including GBM as we described. Two recent meta-analyses identified links between elevated DKC1 expression and poorer survival and presence of metastasis in multiple tumor types (66, 67) while several studies showed that a reduction in DKC1 expression via siRNA or shRNA affects proliferation, migration, invasion, apoptosis, and tumor growth (41, 68–71). Additionally, DKC1 promotes cell immortalization by modulating telomerase activity (12, 69). Agreeing with results showing that certain snoRNAs and modification sites in rRNA are preferentially affected by DKC1 mutations or changes in DKC1 expression levels (40–44), we determined that a partial DKC1 knockdown in GBM cells reduced the levels of a specific group of snoRNAs/scaRNAs. We hypothesize that elevated DKC1 levels increase the production of specific H/ACA snoRNAs and enhance pseudo-uridylation at critical rRNA sites, ultimately altering ribosome composition and function to support tumor progression.

The value of snoRNAs as biomarkers and their potential as targets in cancer therapy have just begun to emerge (72–75). To advance the field, the development of sequencing platforms and dedicated bioinformatics pipelines for accurate quantification and characterization of snoRNAs and scaRNAs, as in this study, is necessary. Targeting “onco-snoRNAs” is still another area under development. Anti-sense oligos (ASOs), which have been successfully used in therapy (76), have been established as the most effective agents to knock down snoRNAs in cells and in tumors (34, 77–81). However, selecting and designing effective snoRNA ASOs remains a challenging task. Due to the limited number of studies, there is insufficient information to choose ASOs based on target sequence motifs, locations, or secondary structure.

The consequences of differential snoRNA expression on rRNA modification and the subsequent impact on translation are just beginning to be explored. Recent studies suggest that variations in the pattern of rRNA modification across tissues, during development, in disease states, and in response to stimuli create a platform for ribosome heterogeneity (10, 82). A notable example comes from a study on *Trypanosoma brucei*. Knocking out a single snoRNA that guides the pseudo-uridylation of nucleotide 530 decreased the presence of eS12 on ribosomes, affecting the translation of mRNAs encoding proteins regulated during the two life stages of the parasite (83). This study also found that rRNA in translating polysomes displays a higher Ψ frequency than total rRNA (83). snoRNAs can regulate additional processes besides rRNA processing and modification. In fact, several snoRNAs known as Orphans are not implicated in rRNA modification and have been shown to interact with chromatin (30, 31, 84). snoRNAs have other non-canonical functions in splicing regulation and RNA silencing (85). Therefore, characterizing targets and the regulatory impact of “onco-snoRNAs” in cancer cells could be challenging.

Fortunately, the advent of novel genomic methods to profile pseudo-uridylation and 2’O methylation (28, 86, 87) as well as platforms to identify snoRNA interactions with other RNA species (29, 88, 89) can greatly facilitate these tasks and advance knowledge on snoRNA function.

## CONCLUSIONS

Using a dedicated Ampliseq platform, we performed the first targeted profiling of snoRNAs and scaRNAs in glioblastoma and glioma stem cells, identifying subsets linked to stemness and differentiation. Our results indicate that changes in snoRNA levels are influenced by both host gene expression and DKC1 activity, suggesting coordinated regulation of snoRNA maturation and function in tumor cells. Elevated DKC1 may enhance the production of specific H/ACA snoRNAs, altering rRNA modification and ribosome composition to support malignancy. These findings highlight the importance of developing specialized sequencing platforms and bioinformatics pipelines for accurate snoRNA quantification, as well as the potential of onco-snoRNAs as biomarkers and therapeutic targets in glioblastoma.

## Supplementary Material

Supplementary Files

This is a list of supplementary files associated with this preprint. Click to download.

- snoRNAs.manuscript.Sup.docx
- TableS1.xlsx
- TableS2.xlsx
- TableS3.xlsx
- TableS4.xlsx
- TableS5.xlsx
- TableS6.xlsx

## Data Availability

The dataset generated during the current study is available in the European Nucleotide Archive repository (https://www.ebi.ac.uk/ena/, under accession PRJEB101101).

## Abbreviations

snoRNAs: small nucleolar RNAs
snoRNPs: small nucleolar ribonucleoprotein complexes
rRNA: ribosomal RNA
GBM: glioblastoma
scaRNAs: Cajal body–specific RNAs
Ampliseq: Amplicon sequencing
GSC: glioma stem cell
DKC1: Dyskerin
FBL: Fibrillarin
Ψ: pseudo-uridine
snRNAs: small nuclear RNAs
TERC: telomerase RNA
lncRNAs: long non-coding RNA
ASOs: antisense oligonucleotides
EGF: epidermal growth factor
FGF: fibroblast growth factor
GFP: Green Fluorescent Protei
RNAseq: RNA sequencing
AST: astrocyte
HBR: human brain
NPC: neuronal precursor cell
ATRA: retinoic acid
CESC: Cervical squamous cell carcinoma and endocervical adenocarcinoma
CHOL: Cholangiocarcinoma
COAD: Colon adenocarcinoma
DLBC: Lymphoid Neoplasm Diffuse Large B-cell Lymphoma
LUSC: Lung squamous cell carcinoma
READ: Rectum adenocarcinoma
STAD: Stomach adenocarcinoma
THYM: Thymoma

## References

[1] Dörner K, Ruggeri C, Zemp I, Kutay U. Ribosome biogenesis factors-from names to functions. EMBO J. 2023;42(7):e112699. 36762427 10.15252/embj.2022112699 PMC10068337

[2] Zisi A, Bartek J, Lindström MS. Targeting ribosome biogenesis in cancer: Lessons learned and way forward. Cancers (Basel). 2022;14(9):2126. 35565259 10.3390/cancers14092126 PMC9100539

[3] Bursać S, Prodan Y, Pullen N, Bartek J, Volarević S. Dysregulated ribosome biogenesis reveals therapeutic liabilities in cancer. Trends Cancer. 2021;7(1):57–76. 32948502 10.1016/j.trecan.2020.08.003

[4] Penzo M, Montanaro L, Treré D, Derenzini M. The ribosome biogenesis-cancer connection. Cells. 2019;8(1):55. 30650663 10.3390/cells8010055 PMC6356843

[5] Genuth NR, Barna M. The discovery of ribosome heterogeneity and its implications for gene regulation and organismal life. Mol Cell. 2018;71(3):364–74. 30075139 10.1016/j.molcel.2018.07.018 PMC6092941

[6] Shi Z, Barna M. Translating the genome in time and space: specialized ribosomes, RNA regulons, and RNA-binding proteins. Annu Rev Cell Dev Biol. 2015;31(1):31–54. 26443190 10.1146/annurev-cellbio-100814-125346

[7] Xue S, Barna M. Specialized ribosomes: a new frontier in gene regulation and organismal biology. Nat Rev Mol Cell Biol. 2012;13(6):355–69. 22617470 10.1038/nrm3359 PMC4039366

[8] Fuentes P, Pelletier J, Gentilella A. Decoding ribosome complexity: role of ribosomal proteins in cancer and disease. NAR Cancer. 2024;6(3):zcae032. 39045153 10.1093/narcan/zcae032 PMC11263879

[9] Gao Y, Wang H. Ribosome heterogeneity in development and disease. Front Cell Dev Biol. 2024;12(1414269):1414269. 39086661 10.3389/fcell.2024.1414269 PMC11288964

[10] Milenkovic I, Novoa EM. Dynamic rRNA modifications as a source of ribosome heterogeneity. Trends Cell Biol. 2025;35(7):604–14. 39500673 10.1016/j.tcb.2024.10.001

[11] Bohnsack MT, Sloan KE. Modifications in small nuclear RNAs and their roles in spliceosome assembly and function. Biol Chem. 2018;399(11):1265–76. 29908124 10.1515/hsz-2018-0205

[12] Garus A, Autexier C. Dyskerin: an essential pseudouridine synthase with multifaceted roles in ribosome biogenesis, splicing, and telomere maintenance. RNA. 2021;27(12):1441–58. 34556550 10.1261/rna.078953.121 PMC8594475

[13] Jorjani H, Kehr S, Jedlinski DJ, Gumienny R, Hertel J, Stadler PF, An updated human snoRNAome. Nucleic Acids Res. 2016;44(11):5068–82. 27174936 10.1093/nar/gkw386 PMC4914119

[14] Kishore S, Gruber AR, Jedlinski DJ, Syed AP, Jorjani H, Zavolan M. Insights into snoRNA biogenesis and processing from PAR-CLIP of snoRNA core proteins and small RNA sequencing. Genome Biol. 2013;14(5):R45. 23706177 10.1186/gb-2013-14-5-r45 PMC4053766

[15] Fafard-Couture É, Jacques PÉ, Scott MS. Motif conservation, stability, and host gene expression are the main drivers of snoRNA expression across vertebrates. Genome Res. 2023;33(4):525–40. 37072185 10.1101/gr.277483.122 PMC10234308

[16] Xiao L, Wang J, Ju S, Cui M, Jing R. Disorders and roles of tsRNA, snoRNA, snRNA and piRNA in cancer. J Med Genet. 2022;59(7):623–31. 35145038 10.1136/jmedgenet-2021-108327

[17] Lv D, Gimple RC, Zhong C, Wu Q, Yang K, Prager BC, PDGF signaling inhibits mitophagy in glioblastoma stem cells through N6-methyladenosine. Dev Cell. 2022;57(12):1466–e816. 35659339 10.1016/j.devcel.2022.05.007 PMC9239307

[18] Mao P, Joshi K, Li J, Kim SH, Li P, Santana-Santos L, Mesenchymal glioma stem cells are maintained by activated glycolytic metabolism involving aldehyde dehydrogenase 1A3. Proc Natl Acad Sci U S A. 2013;110(21):8644–9. 23650391 10.1073/pnas.1221478110 PMC3666732

[19] Eyre TA, Ducluzeau F, Sneddon TP, Povey S, Bruford EA, Lush MJ. The HUGO Gene Nomenclature Database, 2006 updates. Nucleic Acids Res. 2006;34(Database issue):D319–21. 16381876 10.1093/nar/gkj147 PMC1347509

[20] Li H, Handsaker B, Wysoker A, Fennell T, Ruan J, Homer N, The Sequence Alignment/Map format and SAMtools. Bioinformatics. 2009;25(16):2078–9. 19505943 10.1093/bioinformatics/btp352 PMC2723002

[21] Anders S, Pyl PT, Huber W. HTSeq—a Python framework to work with high-throughput sequencing data. Bioinformatics. 2014;31(2):166–9. 25260700 10.1093/bioinformatics/btu638 PMC4287950

[22] Love MI, Huber W, Anders S. Moderated estimation of fold change and dispersion for RNA-seq data with DESeq2. Genome Biol. 2014;15(12):550. 25516281 10.1186/s13059-014-0550-8 PMC4302049

[23] Bray NL, Pimentel H, Melsted P, Pachter L. Near-optimal probabilistic RNA-seq quantification. Nat Biotechnol. 2016;34(5):525–7. 27043002 10.1038/nbt.3519

[24] Trincado JL, Entizne JC, Hysenaj G, Singh B, Skalic M, Elliott DJ, SUPPA2: fast, accurate, and uncertainty-aware differential splicing analysis across multiple conditions. Genome Biol. 2018;19(1):40. 29571299 10.1186/s13059-018-1417-1 PMC5866513

[25] van de Leemput J, Boles NC, Kiehl TR, Corneo B, Lederman P, Menon V, CORTECON: a temporal transcriptome analysis of in vitro human cerebral cortex development from human embryonic stem cells. Neuron. 2014;83(1):51–68. 24991954 10.1016/j.neuron.2014.05.013

[26] Kosti A, Barreiro R, Guardia GDA, Ostadrahimi S, Kokovay E, Pertsemlidis A Synergism of Proneurogenic miRNAs Provides a More Effective Strategy to Target Glioma Stem Cells. Cancers [Internet]. 2021;13(2). Available from: 10.3390/cancers13020289 PMC7831004 33466745

[27] Zhang M, Jiang Z, Ma Y, Liu W, Zhuang Y, Lu B, Quantitative profiling of pseudouridylation landscape in the human transcriptome. Nat Chem Biol. 2023;19(10):1185–95. 36997645 10.1038/s41589-023-01304-7

[28] Xu H, Kong L, Cheng J, Al Moussawi K, Chen X, Iqbal A, Absolute quantitative and base-resolution sequencing reveals comprehensive landscape of pseudouridine across the human transcriptome. Nat Methods. 2024;21(11):2024–33. 39349603 10.1038/s41592-024-02439-8 PMC11541003

[29] Liu B, Wu T, Miao BA, Ji F, Liu S, Wang P, snoRNA-facilitated protein secretion revealed by transcriptome-wide snoRNA target identification. Cell. 2025;188(2):465–e8322. 39579764 10.1016/j.cell.2024.10.046 PMC11761385

[30] Han C, Sun LY, Luo XQ, Pan Q, Sun YM, Zeng ZC, Chromatin-associated orphan snoRNA regulates DNA damage-mediated differentiation via a non-canonical complex. Cell Rep. 2022;38(13):110421. 35354054 10.1016/j.celrep.2022.110421

[31] Yun H, Zoller J, Zhou F, Rohde C, Liu Y, Blank MF, The landscape of RNA-chromatin interaction reveals small non-coding RNAs as essential mediators of leukemia maintenance. Leukemia. 2024;38(8):1688–98. 38942785 10.1038/s41375-024-02322-7 PMC11286530

[32] Zhu H, Wang J, Miao J, Shen M, Wang H, Huang X, SNORD3A regulates STING transcription to promote ferroptosis in acute kidney injury. Adv Sci (Weinh). 2024;11(33):e2400305. 38962954 10.1002/advs.202400305 PMC11434033

[33] Piekna-Przybylska D, Decatur WA, Fournier MJ. The 3D rRNA modification maps database: with interactive tools for ribosome analysis. Nucleic Acids Res. 2008;36(Database issue):D178–83. 17947322 10.1093/nar/gkm855 PMC2238946

[34] Wang Y, An H, Zhang Y, Lyu QR, Zhang Z. SNORA13 antisense oligonucleotides enhances the therapeutical effects of 5-fluorouracil in colon adenocarcinoma. Front Pharmacol. 2025;16:1564682. 40529507 10.3389/fphar.2025.1564682 PMC12171199

[35] Zhou Z, Gu Y, Yi Z, Wang J, Xiong Z, Guo H, SNORA74A drives self-renewal of liver cancer stem cells and hepatocarcinogenesis through activation of Notch3 signaling. Adv Sci (Weinh). 2025;12(26):e2504054. 40270470 10.1002/advs.202504054 PMC12245033

[36] Cui L, Nakano K, Obchoei S, Setoguchi K, Matsumoto M, Yamamoto T, Small nucleolar noncoding RNA SNORA23, up-regulated in human pancreatic ductal adenocarcinoma, regulates expression of spectrin repeat-containing nuclear envelope 2 to promote growth and metastasis of xenograft tumors in mice. Gastroenterology. 2017;153(1):292–e3062. 28390868 10.1053/j.gastro.2017.03.050

[37] Tang Z, Kang B, Li C, Chen T, Zhang Z. GEPIA2: an enhanced web server for large-scale expression profiling and interactive analysis. Nucleic Acids Res. 2019;47(W1):W556–60. 31114875 10.1093/nar/gkz430 PMC6602440

[38] Fafard-Couture É, Bergeron D, Couture S, Abou-Elela S, Scott MS. Annotation of snoRNA abundance across human tissues reveals complex snoRNA-host gene relationships. Genome Biol. 2021;22(1):172. 34088344 10.1186/s13059-021-02391-2 PMC8176728

[39] Bouchard-Bourelle P, Desjardins-Henri C, Mathurin-St-Pierre D, Deschamps-Francoeur G, Fafard-Couture É, Garant JM, snoDB: an interactive database of human snoRNA sequences, abundance and interactions. Nucleic Acids Res. 2020;48(D1):D220–5. 31598696 10.1093/nar/gkz884 PMC6943035

[40] Taoka M, Nobe Y, Yamaki Y, Sato K, Ishikawa H, Izumikawa K, Landscape of the complete RNA chemical modifications in the human 80S ribosome. Nucleic Acids Res. 2018;46(18):9289–98. 30202881 10.1093/nar/gky811 PMC6182160

[41] Guerrieri AN, Zacchini F, Onofrillo C, Di Viggiano S, Penzo M, Ansuini A, DKC1 Overexpression induces a more aggressive cellular behavior and increases intrinsic ribosomal activity in immortalized mammary gland cells. Cancers (Basel). 2020;12(12):3512. 33255756 10.3390/cancers12123512 PMC7760958

[42] Barozzi C, Zacchini F, Corradini AG, Morara M, Serra M, De Sanctis V, Alterations of ribosomal RNA pseudouridylation in human breast cancer. NAR Cancer. 2023;5(2):zcad026. 37260601 10.1093/narcan/zcad026 PMC10227372

[43] Montanaro L, Brigotti M, Clohessy J, Barbieri S, Ceccarelli C, Santini D, Dyskerin expression influences the level of ribosomal RNA pseudo-uridylation and telomerase RNA component in human breast cancer. J Pathol. 2006;210(1):10–8. 16841302 10.1002/path.2023

[44] Bellodi C, McMahon M, Contreras A, Juliano D, Kopmar N, Nakamura T, H/ACA small RNA dysfunctions in disease reveal key roles for noncoding RNA modifications in hematopoietic stem cell differentiation. Cell Rep. 2013;3(5):1493–502. 23707062 10.1016/j.celrep.2013.04.030 PMC3857015

[45] Guglas K, Kozłowska-Masłoń J, Kolenda T, Paszkowska A, Teresiak A, Bliźniak R, Midsize noncoding RNAs in cancers: a new division that clarifies the world of noncoding RNA or an unnecessary chaos? Rep Pract Oncol Radiother. 2022;27(6):1077–93. 36632289 10.5603/RPOR.a2022.0123 PMC9826665

[46] Cao T, Rajasingh S, Samanta S, Dawn B, Bittel DC, Rajasingh J. Biology and clinical relevance of noncoding sno/scaRNAs. Trends Cardiovasc Med. 2018;28(2):81–90. 28869095 10.1016/j.tcm.2017.08.002 PMC5762389

[47] Patil P, Kibiryeva N, Uechi T, Marshall J, O’Brien JE Jr, Artman M, scaRNAs regulate splicing and vertebrate heart development. Biochim Biophys Acta. 2015;1852(8):1619–29. 25916634 10.1016/j.bbadis.2015.04.016

[48] Beneventi G, Munita R, Cao Thi Ngoc P, Madej M, Cieśla M, Muthukumar S, The small Cajal body-specific RNA 15 (SCARNA15) directs p53 and redox homeostasis via selective splicing in cancer cells. NAR Cancer. 2021;3(3):zcab026. 34316713 10.1093/narcan/zcab026 PMC8271217

[49] Song J, Zheng A, Li S, Zhang W, Zhang M, Li X, Clinical significance and prognostic value of small nucleolar RNA SNORA38 in breast cancer. Front Oncol. 2022;12:930024. 36158687 10.3389/fonc.2022.930024 PMC9500313

[50] Schulten HJ, Bangash M, Karim S, Dallol A, Hussein D, Merdad A, Comprehensive molecular biomarker identification in breast cancer brain metastases. J Transl Med. 2017;15(1):269. 29287594 10.1186/s12967-017-1370-x PMC5747948

[51] Korsgaard U, García-Rodríguez JL, Jakobsen T, Ahmadov U, Dietrich KG, Vissing SM, The transcriptional landscape of coding and noncoding RNAs in recurrent and nonrecurrent colon cancer. Am J Pathol. 2024;194(8):1424–42. 38704091 10.1016/j.ajpath.2024.04.003

[52] Li S, Jin Z, Song X, Ma J, Peng Z, Yu H, The small nucleolar RNA SNORA51 enhances breast cancer stem cell-like properties via the RPL3/NPM1/c-MYC pathway. Mol Carcinog. 2024;63(6):1117–32. 38421204 10.1002/mc.23713

[53] Gómez-Matas J, Duran-Sanchon S, Lozano JJ, Ferrero G, Tarallo S, Pardini B, SnoRNA profiling in colorectal cancer and assessment of non-invasive biomarker capacity by ddPCR in fecal samples. iScience. 2024;27(3):109283. 38450150 10.1016/j.isci.2024.109283 PMC10915595

[54] Yu L, Zhang M, Ma Z, Wu S. Expression of small nucleolar RNA SNORA51 and its clinical significance in hepatocellular carcinoma. Oncol Lett. 2024;27(2):55. 38192654 10.3892/ol.2023.14188 PMC10773229

[55] Wang LY, Song JN, Chen YX, Zhu Y, Ren HL, Li QQ, Characterization the prognosis role and effects of snoRNAs in melanoma patients. Exp Dermatol. 2024;33(1):e14944. 37772659 10.1111/exd.14944

[56] Huang R, Liao X, Li Q. Integrative genomic analysis of a novel small nucleolar RNAs prognostic signature in patients with acute myelocytic leukemia. Math Biosci Eng. 2022;19(3):2424–52. 35240791 10.3934/mbe.2022112

[57] Feng S, Zhang X, Gu X, Zhou M, Chen Y, Wang C. Identification of six novel prognostic gene signatures as potential biomarkers in small cell lung cancer. Comb Chem High Throughput Screen. 2023;26(5):938–49. 35490316 10.2174/1386207325666220427121619

[58] Tang G, Zeng Z, Sun W, Li S, You C, Tang F, Small nucleolar RNA 71A promotes lung cancer cell proliferation, migration and invasion via MAPK/ERK pathway. J Cancer. 2019;10(10):2261–75. 31258730 10.7150/jca.31077 PMC6584411

[59] Ding Y, Sun Z, Zhang S, Zhou L, Xu Q, Zhou D, Identification of snoRNA SNORA71A as a novel biomarker in prognosis of hepatocellular carcinoma. Dis Markers. 2020;2020:8879944. 33062075 10.1155/2020/8879944 PMC7537701

[60] Xu M, Meng Y, Li Q, Charwudzi A, Qin H, Xiong S. Identification of biomarkers for early diagnosis of multiple myeloma by weighted gene co-expression network analysis and their clinical relevance. Hematology. 2022;27(1):322–31. 35231203 10.1080/16078454.2022.2046326

[61] Zhang Z, Tao Y, Hua Q, Cai J, Ye X, Li H. SNORA71A promotes colorectal cancer cell proliferation, migration, and invasion. Biomed Res Int. 2020;2020(1):8284576. 33083486 10.1155/2020/8284576 PMC7559222

[62] Hu T, Lu C, Xia Y, Wu L, Song J, Chen C, Small nucleolar RNA SNORA71A promotes epithelialmesenchymal transition by maintaining ROCK2 mRNA stability in breast cancer. Mol Oncol. 2022;16(9):1947–65. 35100495 10.1002/1878-0261.13186 PMC9067147

[63] Gong J, Li Y, Liu CJ, Xiang Y, Li C, Ye Y, A pan-cancer analysis of the expression and clinical relevance of small nucleolar RNAs in human cancer. Cell Rep. 2017;21(7):1968–81. 29141226 10.1016/j.celrep.2017.10.070

[64] Chen JL, Leeder WM, Morais P, Adachi H, Yu YT. Pseudouridylation-mediated gene expression modulation. Biochem J. 2024;481(1):1–16. 38174858 10.1042/BCJ20230096

[65] Borchardt EK, Martinez NM, Gilbert WV. Regulation and function of RNA pseudouridylation in human cells. Annu Rev Genet. 2020;54(1):309–36. 32870730 10.1146/annurev-genet-112618-043830 PMC8007080

[66] Zhang Q, Liu X, Zou Z, Zhou B. Evidence from a meta-analysis for the prognostic and clinicopathological importance of DKC1 in malignancies. Future Oncol. 2023;19(6):473–84. 36876511 10.2217/fon-2022-1125

[67] Liu XY, Tan Q, Li LX. A pan-cancer analysis of Dyskeratosis congenita 1 (DKC1) as a prognostic biomarker. Hereditas. 2023;160(1):38. 38082360 10.1186/s41065-023-00302-y PMC10712082

[68] Kan G, Wang Z, Sheng C, Chen G, Yao C, Mao Y, Dual inhibition of DKC1 and MEK1/2 synergistically restrains the growth of colorectal cancer cells. Adv Sci (Weinh). 2021;8(10):2004344. 34026451 10.1002/advs.202004344 PMC8132060

[69] Kan G, Wang Z, Sheng C, Yao C, Mao Y, Chen S. Inhibition of DKC1 induces telomere-related senescence and apoptosis in lung adenocarcinoma. J Transl Med. 2021;19(1):161. 33879171 10.1186/s12967-021-02827-0 PMC8056518

[70] Hou P, Shi P, Jiang T, Yin H, Chu S, Shi M, DKC1 enhances angiogenesis by promoting HIF-1α transcription and facilitates metastasis in colorectal cancer. Br J Cancer. 2020;122(5):668–79. 31857720 10.1038/s41416-019-0695-z PMC7054532

[71] Miao FA, Chu K, Chen HR, Zhang M, Shi PC, Bai J, Increased DKC1 expression in glioma and its significance in tumor cell proliferation, migration and invasion. Invest New Drugs. 2019;37(6):1177–86. 30847721 10.1007/s10637-019-00748-w

[72] Yan X, Chen B, Song X, Zhou Y, Jin F, Zheng A. Mechanisms of snoRNAs in cancer treatment resistance: from molecular insights to clinical applications. Trends Genet [Internet]. 2025; Available from: 10.1016/j.tig.2025.06.004 40651850

[73] Faucher-Giguère L, de Préval BS, Rivera A, Scott MS, Elela SA. Small nucleolar RNAs: the hidden precursors of cancer ribosomes. Philos Trans R Soc Lond B Biol Sci. 2025;380(1921):20230376. 40045787 10.1098/rstb.2023.0376 PMC11883439

[74] Hu X, Cui W, Liu M, Zhang F, Zhao Y, Zhang M, SnoRNAs: The promising targets for anti-tumor therapy. J Pharm Anal. 2024;14(11):101064. 39634568 10.1016/j.jpha.2024.101064 PMC11613181

[75] Zacchini F, Barozzi C, Venturi G, Montanaro L. How snoRNAs can contribute to cancer at multiple levels. NAR Cancer. 2024;6(1):zcae005. 38406265 10.1093/narcan/zcae005 PMC10894041

[76] Naeem S, Zhang J, Zhang Y, Wang Y. Nucleic acid therapeutics: Past, present, and future. Mol Ther Nucleic Acids. 2025;36(1):102440. 39897578 10.1016/j.omtn.2024.102440 PMC11786870

[77] Liang XH, Vickers TA, Guo S, Crooke ST. Efficient and specific knockdown of small non-coding RNAs in mammalian cells and in mice. Nucleic Acids Res. 2011;39(3):e13. 21062825 10.1093/nar/gkq1121 PMC3035437

[78] Ideue T, Hino K, Kitao S, Yokoi T, Hirose T. Efficient oligonucleotide-mediated degradation of nuclear noncoding RNAs in mammalian cultured cells. RNA. 2009;15(8):1578–87. 19535462 10.1261/rna.1657609 PMC2714749

[79] Liang XH, Sun H, Nichols JG, Crooke ST. RNase H1-dependent antisense oligonucleotides are robustly active in directing RNA cleavage in both the cytoplasm and the nucleus. Mol Ther. 2017;25(9):2075–92. 28663102 10.1016/j.ymthe.2017.06.002 PMC5589097

[80] Chen S, Wen JT, Zhang S, Wang JL, Yuan J, Bao HJ, SNORD9 promotes ovarian cancer tumorigenesis via METTL3/IGF2BP2-mediated NFYA m6A modification and is a potential target for antisense oligonucleotide therapy. Life Sci. 2025;368:123527. 40044032 10.1016/j.lfs.2025.123527

[81] Chen S, Li QH, Chen X, Bao HJ, Wu W, Shen F, SNORA70E promotes the occurrence and development of ovarian cancer through pseudouridylation modification of RAP1B and alternative splicing of PARPBP. J Cell Mol Med. 2022;26(20):5150–64. 36056690 10.1111/jcmm.17540 PMC9575132

[82] Henras AK, Plisson-Chastang C, Humbert O, Romeo Y, Henry Y. Synthesis, function, and heterogeneity of snoRNA-guided posttranscriptional nucleoside modifications in eukaryotic ribosomal RNAs. Enzymes. 2017;41:169–213. 28601222 10.1016/bs.enz.2017.03.007

[83] Rajan KS, Madmoni H, Bashan A, Taoka M, Aryal S, Nobe Y, A single pseudouridine on rRNA regulates ribosome structure and function in the mammalian parasite Trypanosoma brucei. Nat Commun. 2023;14(1):7462. 37985661 10.1038/s41467-023-43263-6 PMC10662448

[84] Jinn S, Brandis KA, Ren A, Chacko A, Dudley-Rucker N, Gale SE, snoRNA U17 regulates cellular cholesterol trafficking. Cell Metab. 2015;21(6):855–67. 25980348 10.1016/j.cmet.2015.04.010 PMC4456254

[85] Wajahat M, Bracken CP, Orang A. Emerging functions for snoRNAs and snoRNA-derived fragments. Int J Mol Sci. 2021;22(19):10193. 34638533 10.3390/ijms221910193 PMC8508363

[86] Tang Y, Wu Y, Wang S, Lu X, Gu X, Li Y, An integrative platform for detection of RNA 2’-O-methylation reveals its broad distribution on mRNA. Cell Rep Methods. 2024;4(3):100721. 38452769 10.1016/j.crmeth.2024.100721 PMC10985248

[87] Chen L, Zhang LS, Ye C, Zhou H, Liu B, Gao B, Nm-Mut-seq: a base-resolution quantitative method for mapping transcriptome-wide 2’-O-methylation. Cell Res. 2023;33(9):727–30. 37316584 10.1038/s41422-023-00836-w PMC10474006

[88] Deschamps-Francoeur G, Couture S, Abou-Elela S, Scott MS. The snoGloBe interaction predictor reveals a broad spectrum of C/D snoRNA RNA targets. Nucleic Acids Res. 2022;50(11):6067–83. 35657102 10.1093/nar/gkac475 PMC9226514

[89] Song Z, Bae B, Schnabl S, Yuan F, De Zoysa T, Akinyi MV, Mapping snoRNA-target RNA interactions in an RNA-binding protein-dependent manner with chimeric eCLIP. Genome Biol. 2025;26(1):39. 40001124 10.1186/s13059-025-03508-7 PMC11863803

